# Characterization of the YdeO Regulon in *Escherichia coli*

**DOI:** 10.1371/journal.pone.0111962

**Published:** 2014-11-06

**Authors:** Yuki Yamanaka, Taku Oshima, Akira Ishihama, Kaneyoshi Yamamoto

**Affiliations:** 1 Department of Frontier Bioscience, Hosei University, Koganei, Tokyo, Japan; 2 Micro-Nano Technology Research Center, Hosei University, Koganei, Tokyo, Japan; 3 Graduate School of Information Sciences, Nara Institute of Science and Technology, Ikoma, Nara, Japan; Indian Institute of Science, India

## Abstract

Enterobacteria are able to survive under stressful conditions within animals, such as acidic conditions in the stomach, bile salts during transfer to the intestine and anaerobic conditions within the intestine. The glutamate-dependent (GAD) system plays a major role in acid resistance in *Escherichia coli*, and expression of the GAD system is controlled by the regulatory cascade consisting of EvgAS > YdeO > GadE. To understand the YdeO regulon *in vivo*, we used ChIP-chip to interrogate the *E. coli* genome for candidate YdeO binding sites. All of the seven operons identified by ChIP-chip as being potentially regulated by YdeO were confirmed as being under the direct control of YdeO using RT-qPCR, EMSA, DNaseI-footprinting and reporter assays. Within this YdeO regulon, we identified four stress-response transcription factors, DctR, NhaR, GadE, and GadW and enzymes for anaerobic respiration. Both GadE and GadW are involved in regulation of the GAD system and NhaR is an activator for the sodium/proton antiporter gene. In conjunction with co-transcribed Slp, DctR is involved in protection against metabolic endoproducts under acidic conditions. Taken all together, we suggest that YdeO is a key regulator of *E. coli* survival in both acidic and anaerobic conditions.

## Introduction

Enterobacteria such as *Escherichia coli*, exist in the environment, and in the gut of warm blooded animals. To survive this switch in lifestyles, and upon ingestion by a new host, bacteria are directly exposed to various stresses and hence require sophisticated stress response systems to survive continuous changes in environment such as acidic conditions in the stomach, bile salts, and anaerobic conditions within the intestines [1]. For survival under acidic conditions, *E. coli* possesses three amino acid-dependent acid resistance systems with glutamate, arginine, and lysine [2], [3], [4]. The resistance mechanism involves the transient consumption of the intracellular proton by glutamate, arginine and lysine decarboxylases, and exchange of the amine products with extracellular amino acids through their respective antiporters [2], [3], [5], [6]. The most effective system of acid resistance is the GAD (glutamic acid-dependent) system which is composed of two glutamate decarboxylase isozymes, GadA and GadB, and the cognate antiporter GadC. Expression of these components is under the control of a complex network of transcription factors, including GadE, GadX, GadW, EvgA, YdeO, and H-NS [1].

YdeO is a transcription factor, belonging to the AraC/XylS family. Knowledge about the regulatory functions of YdeO is limited except that it is known that YdeO activates transcription of the *gad* system components, *gadE*, *gadA* and *gadBC* [7], [8], [9]. The expression of *ydeO* is activated by the two-component system EvgSA [9], [10], [11], forming a regulatory cascade, EvgA > YdeO > GadE [9], [12]. In this study, we performed a comprehensive interrogation of YdeO-binding sites *in vivo* on the *E. coli* genome using ChIP-chip analysis, and identified a set of YdeO-regulated genes, including four stress-response transcription factors, DctR, NhaR, GadE, and GadW, and several genes involved in respiration. Taking these observations together we propose that YdeO is the regulator which coordinates the response to acid and anaerobic conditions in *E. coli*.

## Materials and Methods

### *E. coli* strains and growth conditions

*E. coli* strains and plasmids used in this study are shown in Table S1. *E. coli* cells were grown at 37°C in Luria-Bertani (LB) medium. Cell growth was monitored by measuring the turbidity with a Mini photo 518R spectrophotometer (Taitec). The standard procedure for bacterial cell cultivation in this study was as follows: A single colony was isolated from an overnight culture on a LB agar plate, and inoculated into 5 ml of fresh LB medium. This liquid culture was grown overnight at 37°C, and the overnight culture was diluted 100-fold into fresh LB medium. The culture was incubated at 37°C with reciprocal shaking (160 revolutions min^−1^) for aerobiosis or without shaking for anaerobiosis.

### Introduction of a tagged gene into the *E. coli* genome

The introduction of a tagged gene into the *E. coli* genome was carried out using the method of Uzzau et al. [13]. In brief, primers were used to make PCR extensions homologous to the last portion of the targeted gene (forward primer) and to a region downstream of it (reverse primer) as follows; YDEOF-1 (forward) and YDEOR-1 (reverse) for *ydeO*-*3xflag*; GADE-F (forward) and GADE-R (reverse) for *gadE-3xflag*; GADW-F (forward) and GADW-R (reverse) for *gadW-3xflag* (Table S1). Amplified DNA fragments including the 3′ sequence with flag tag and a kanamycin-resistance gene were amplified by PCR using pSUB11 as a template, a pair of primers, and Ex-Taq DNA polymerase (Takara Bio). PCR products were purified using a QIAquick PCR purification kit (Qiagen), and then used directly for electro-transformation. *E. coli* carrying a lambda-Red helper plasmid, pKD46, was used to make competent cells, and were grown at 30°C in LB medium supplemented with 100 µg ml^−1^ ampicillin and 1 mM arabinose to an OD_600_ of 0.4. Cells were collected by centrifugation, and washed two times with ice-cold sterile deionized water containing 10% glycerol. Aliquots (50 µl) of the bacterial suspensions in 10% glycerol were mixed with more than 1 µg of PCR product in a chilled cuvette (0.2 cm electrode gap) and subjected to a single pulse (2.5 kV) by a Gene pulser Xcell (Bio Rad). After 1 hr recovery at 37°C in 1 mL of SOC medium (2% tryptone, 0.5% yeast extract, 10 mM NaCl, 2.5 mM KCl, 10 mM MgCl_2_, 10 mM MgSO_4_, 20 mM glucose) containing 1 mM arabinose, half of the volume of electroporated bacteria in SOC media were spread on to LB agar plates supplemented with antibiotics for the selection of kanamycin-resistant recombinants. If none grew on the agar plate after incubation overnight at 37°C, the remainder stored was spread on to LB kan plates. The kanamycin-resistance recombinants were isolated once on LB agar at 37°C, and then examined for ampicillin sensitivity for loss of the helper plasmid.

### Construction of YdeO expression plasmids

To construct pYY0401 for YdeO-3xFLAG expression, DNA fragments containing the *ydeO* coding region were amplified by PCR using *E. coli* YY5001 genomic DNA, including the *3xflag* tag at the end of *ydeO* as a template, and a pair of primers, YDEOF-2 and YDEOR-3, in which the *Bam* HI and *Eco* RI sites were included (see sequences in Table. S1). After digestion of PCR products with *Bam* HI and *Eco* RI, the PCR-amplified fragments were cloned into the pTrc99A vector containing an inducible *trc* promoter between the *Bam* HI and *Eco* RI sites. To construct pYdeO for expression of intact YdeO, DNA fragments containing the *ydeO* coding region were amplified by PCR using *E. coli* W3110 type A [14] genomic DNA as a template and the primers, YDEOF-2 and YDEOR-2 (see sequences in Table. S1). After digestion of the PCR product with *Bam* HI and *Eco* RI, the PCR-amplified fragments were ligated into the pTrc99A vector between appropriate restriction enzyme sites. To construct pYdeO-SUMO for overproduction of SUMO (Small Ubiquitin-related MOdifier) fused YdeO, DNA fragments containing the *ydeO* coding region were amplified by PCR using *E. coli* BW25113 genomic DNA as a template and the primers, YDEO-SUMO-F and YDEO-SUMO-R, in which 15-nt homologous to pE-SUMO vector (Life Sensors) digested with *Bsa* I were included (see sequences in Table. S1). The PCR-amplified fragments were cloned into the pE-SUMO vector using In-Fusion HD cloning kit (Clontech). All of the plasmids were confirmed by DNA sequencing with primers, Trc99A-F and/or Trc99A-R for pTrc99A derivatives and T7 terminator and SUMO forward for pE-SUMO derivatives.

### Construction of *lacZ* and *lux* reporter plasmids

To construct a *lacZ* fusion gene, the pRS552 plasmid was used as a vector for the construction of translational fusions [15]. The promoter DNA fragment was amplified by PCR using the genome of *E. coli* W3110 type-A strain [14] as a template and a pair of primers. The primers used were: APPC-LF and APPC-LR for pAPPC-L; YIIS-LF and YIIS-LR for pYY0503; HYAA-LF and HYAA-LR for pHYAA-L (Table S1). The PCR product was digested with *BamH* I and/or *EcoR* I and then ligated into pRS552 at the corresponding sites. A *nhaR-lux* transcription fusion was also constructed. First, DNA fragments containing the *nhaR* promoter were amplified by PCR using the primers: NHAR-lux-F and NHA-lux-R, which contained 15-nt homologous to the pLUX vector [16] digested with *Xho* I and *Bam* HI were included (see sequences in Table. S1). The PCR-amplified fragments were cloned into the pLUX vector using In-Fusion HD cloning kit (Clontech), resulting in the construction of pLUXnhaR (Table S1). All of the plasmids were confirmed by DNA sequencing using the lacZ-30R primer complementary to *lacZ* or Lux-R primer complementary to *luxC* in a vector.

### ChIP-chip analysis

The ChIP-chip assay was carried out as described in previous reports [17], [18], [19] with a few modifications. YY0201 (Δ*ydeO*) harbouring pYY0401 (*ydeO*-*3xflag*) was grown to an OD_600_ of 0.4 then re-incubated in LB medium containing formaldehyde (final concentration of 1%) at 37°C for 30 min. The cross-linking reaction was terminated by the addition of glycine, and cells were collected, washed, re-suspended with lysis buffer, and lysed by incubation with Lysozyme. Lysed cells were dissolved in 4 ml of IP buffer containing PMSF. The sample was then sonicated 60 times for 30 sec at 30 sec intervals on ice using a BRANSON Digital Sonifier (Branson). After centrifugation, the supernatant fraction (whole cell extract) was mixed with anti-FLAG antibody (Sigma Aldrich)-coated-protein A Dynal Dynabeads (Invitrogen) and incubated at 4°C overnight. After washing twice with IP buffer and IP salt buffer, the DNA–YdeO-3xFLAG complex bound to the beads was recovered by eluting with elution buffer (50 mM Tris–HCl pH 7.5, 10 mM EDTA, 1% SDS). YdeO-3xFLAG in whole cell extracts and in immunoprecipitated DNA fractions were digested by Pronase (Roche). DNA fragments free of cross-linked DNA–protein were purified using a QIAquick PCR purification kit (Qiagen). Recovered DNA fragments were amplified according to the random DNA amplification method using the primers, PF 43 and PF 44 described by Katou et al. [17]. PCR was performed over 30 cycles, using Phusion high-fidelity DNA polymerase (New England Biolabs). Amplified DNA fragments were terminally labeled and hybridized with the custom-designed Affymetrix oligonucleotide tiling array and raw data (CEL files) were processed using the Array edition of the In Silico Molecular Cloning (IMC) software (In Silico Biology) as previously described [18], [19], [20]. To detect DNA fragments by immunoprecipitation, the signal intensities of ChIP DNA were divided by those of the supernatant (Sup) fraction.

### Pufirication of the YdeO protein

In a typical procedure [21], a single colony of transformed *E. coli* BL21 (DE3) was grown to OD_600_ = 0.6 at 37°C with shaking in LB medium supplemented with 100 µg ml^−1^ ampicillin. The culture was then cooled on ice, induced with 4.5 mM IPTG, and incubated at 20°C overnight with shaking. Cells were isolated by centrifugation and resuspended in 400 µL of lysis buffer (100 mM NaCl, 50 mM Tris-HCl pH 8.0) containing 0.2 mM PMSF. Cells were treated with lysozyme and then subjected to sonication. Triton X-100 was added to 1% (v/v) and incubated on ice for 1 hr. The culture was centrifuged, and the supernatant was decanted and stored at 4°C. Supernatant was mixed with 2 ml of 50% Ni-nitrilotriacetic acid (NTA) agarose solution (Qiagen) and loaded onto a column. The column was washed with 10 ml of lysis buffer containing 1%Triton X-100, and then washed with 10 ml of lysis buffer containing 1%Triton X-100 and 25 mM imidazole. Proteins were eluted with 3 ml of each elution buffer (lysis buffer containing 1%Triton x-100 and 0.1 M, 0.2 M, 0.3 M, 0.4 M, or 0.5 M imidazole), and peak fractions of transcription factors were pooled and dialyzed against a storage buffer (50 mM Tris-HCl, pH 7.5 at 4°C, 200 mM KCl, 10 mM MgCl_2_, 0.1 mM EDTA, 5 mM DTT, and 50% glycerol), and stored at –80°C until use. Protein purity was checked on SDS-PAGE.

### Preparation of total RNA from *E. coli* cells

Total RNA was prepared using the as previously described [22]. A single colony of *E. coli* was grown in LB medium to OD_600_ = 0.3 at 37°C with shaking. Cells were harvested and total RNAs were prepared using hot phenol. In brief, total RNA was extracted with H_2_O-saturated phenol and precipitated with ethanol. After digestion with RNase-free DNase I (Takara Bio), total RNA was extracted with H_2_O-saturated phenol and precipitated with ethanol, and dissolved in RNase-free water. The concentration of total RNA was determined by measuring the absorbance at 260 nm. The purity of total RNA was checked by agarose gel electrophoresis.

### Transcriptome analysis

To prepare fluorescently labeled cDNA, total RNA (5 µg) was used. We used the FairPlay III Microarray Labeling kit (Agilent), CyDye Cy3 mono-reactive Dye, and CyDye Cy5 mono-reactive Dye (GE Healthcare). For all experiments, two sets of RNAs from an independent colony were carried out with a pair of the fluorescence dye. The mixture containing 1 µl of Ramdom hexanucleotide primers, 5 µg of total RNA, and 12 µl of DEPC-treated water was heated at 75°C for 10 min and cooled to room temperature. After addition of 3 µl of Affinity script HC RTase (Agilent), 1X Affinity script RT buffer, 1X dNTP mixture, 75 mM DTT, and 0.5 µl of RNase block to 10 µl of RNA/primer mixture product, cDNA synthesis was carried out at 42°C for 1 hr and stopped by addition of 10 µM NaOH. The mixture was neutralized by addition of 10 µM HCl. The synthesized cDNA was purified by ethanol-precipitation and then labelled by CyDye Cy3 mono-reactive Dye or CyDye Cy5 mono-reactive Dye. The dye-coupled cDNA was purified by attached the micro spin cup.

The *E. coli* Gene Expression Microarray microarray 8×15 K (Agilent) was used. Each 300 ng of Cy3- and Cy5-labeled cDNA were mixed and added to 1X Blocking Buffer (Agilent) and 1X HI-RPM GE Hybridization Buffer (Agilent). After precipitation of impurities, 40 µl of the labelled-cDNA mixture was applied to the DNA chip, and the hybridization was carried out at 65°C for 17 hr. The DNA chip was washed at room temperature with Agilent Gene Expression Wash Buffer 1 (Agilent) and at 37°C with Agilent Gene Expression Wash Buffer 2 (Agilent). The DNA chip was scanned with an Agilent G2565CA microarray scanner Ver. 8.1, and the intensities of both Cy3 and Cy5 were quantified by Feature Extraction Ver. 8.1. And then, the Cy5/Cy3 ratios were calculated from the normalized values.

### RT-qPCR

Total RNAs were transcribed to cDNA with random primers using Primer Script 1^st^ strand cDNA synthesis Kit (Takara Bio). Quantitative PCR (qPCR) was conducted using SYBR Green PCR Master Mix (Applied Biosystems). Pairs of primers used are described in Table S1. The cDNA templates were twofold serially diluted and used in the qPCR assays. The qPCR reaction mixtures, each containing 12.5 µl of 2X Power SYBR Green PCR Master Mix (Applied Biosystems), 0.225 µl of each primer (10 µM stock), 9.55 µl of water, and 2.5 µl of cDNA, were amplified under the following thermal cycle conditions of: 50°C for 2 min and 95°C for 10 min followed by 40 cycles of 15 sec at 95°C and then 60 sec at 60°C. The expression levels of the 16 S rRNA gene were used for normalization of data, and the relative expression levels were quantified using ‘Delta–delta method’ presented by PE Applied Biosystems (Perkin Elmer) as described in previous reports [23], [24]. The results presented are averages of the results from the replicate experiments ± standard errors of the means (SEM).

### EMSA

Probes were amplified by PCR using the previously constructed reporter plasmids as templates, with a pair of primers: a specific primer and an FITC-labeled primer. PCR products with FITC at their termini were purified using the QIAquick PCR purification kit (Qiagen). For gel shift assays, mixtures of the FITC-labeled probes and purified SUMO-YdeO were incubated at 37°C for 30 min in gel shift buffer (50 mM Tris-HCl, pH 7.8 at 37°C, 50 mM NaCl, 3 mM Mg acetate, 0.1 mM EDTA, 0.1 mM DTT, and 0.37 µM BSA) containing 0.2 µg ml^−1^ salmon sperm DNA. After addition of a DNA dye solution, the mixture was directly subjected to 4% or 7% PAGE. Fluorescent-labeled DNA in gels was detected using Typhoon 9410 (Amersham Biosciences).

### DNase I footprinting analysis

The probe was amplified by PCR using a pLUXgadWp as a template, primer pairs GADW-F-2 and Lux-R-FITC, and Ex Taq DNA polymerase (Takara). 1.0 pmol of a FITC-labeled probe was incubated at 37°C for 30 min with purified SUMO-YdeO (0.5 to 15 pmol) in 25 µl of gel shift buffer (50 mM Tris-HCl, pH 7.8 at 37°C, 50 mM NaCl, 3 mM Mg acetate, 0.1 mM EDTA, 0.1 mM DTT, and 0.37 µM BSA). After incubation for 30 min, DNA was digested by DNase I (Takara Bio) for 30 s at 25°C, and then the reaction was terminated by addition of phenol. DNA was precipitated by ethanol, dissolved in formamide dye solution, and analyzed by electrophoresis on a DNA analyzer DSQ-2000L (Shimadu).

### Measurement of luciferase activity in *E. coli*

A single colony of a strain freshly transformed with one of the luciferase reporter plasmids (Table S1) was grown in LB medium supplemented with 50 µg ml^−1^ kanamycin to OD_600_ = 0.3 at 37°C with shaking. At this point, the culture was transferred to a micro-titer plate (96-well micro-titer) to start monitoring reporter activity measurement in an automated plate reader MTP-880 (Corona). The Lux (luciferase activity) reads were then divided by the equivalent OD reads (Lux/OD) to approximate Lux activity unit per cell mass for each well. The Lux/OD values of the three technical replicate wells of each culture were averaged.

### Measurement of β-galactosidase activity in *E. coli*

*E. coli* cells were grown in LB medium and subjected to measurement of β-galactosidase activity with *o*-nitrophenyl-D-galactopyranoside as described in the previous report [11].

### Western blotting analysis

*E. coli* cells grown in LB medium were harvested by centrifugation and re-suspended in 0.4 ml lysis buffer containing 8 M urea and sonicated. After centrifugation, the same volume of supernatant was subjected to 15% SDS-PAGE and blotted on to PVDF membranes using an iBlot semi-dry transfer apparatus (Invitrogen). Membranes were first immuno-detected with anti-FLAG (Sigma), anti-NhaR serum (Lab stock), or anti-α (Neoclone) and HRP-conjugated anti-mouse IgG (Nacalai Tesque) antibodies and then developed with a chemiluminescence kit (Nacalai Tesque). The image was analyzed with a LAS-4000 IR multi colour imager (Fuji Film).

## Results

### Identification of YdeO associated sites *in vivo* within the *E. coli* genome

To identify the genes directly regulated by YdeO, we first determined the genome-wide distribution of YdeO-binding sites by ChIP-chip (Chromatin ImmunoPreciptation-DNA chip) analysis. For this purpose, we inserted a *3xflag* tail into the 3′ end of the *ydeO* gene in the genome and tried to prepare YdeO-DNA complexes for ChIP-chip analysis from the YY5001 strain harbouring *ydeO*-*3xflag* grown in LB medium at 37°C with shaking. The level of YdeO-3xFLAG expression was, however, not enough to isolate YdeO-DNA complexes using the anti-FLAG antibody. We then constructed plasmid pYY0401 for the expression of YdeO-3xFLAG and transformed it into the *ydeO*-deficient mutant. The *ydeO*-deficient mutant transformed with pYY0401 was grown until it reached log phase and was then treated with formaldehyde for DNA-protein cross-linking. The *E. coli* cells were disrupted with sonication to prepare a whole cell extract from which YdeO-DNA complexes were isolated, sonicated and subjected to immune-precipitation using anti-FLAG antibody. After the pronase treatment, ChIP DNA fragments were isolated from the YdeO-DNA complexes for mapping on the genome. As an internal reference for the specific binding of YdeO with its targets, we interrogated the association of YdeO with the *gadE* promoter, the only known target of YdeO. After PCR amplification from the ChIP DNA samples using specific primers, the *gadE* promoter could be specifically amplified (data not shown).

**Figure 1:**
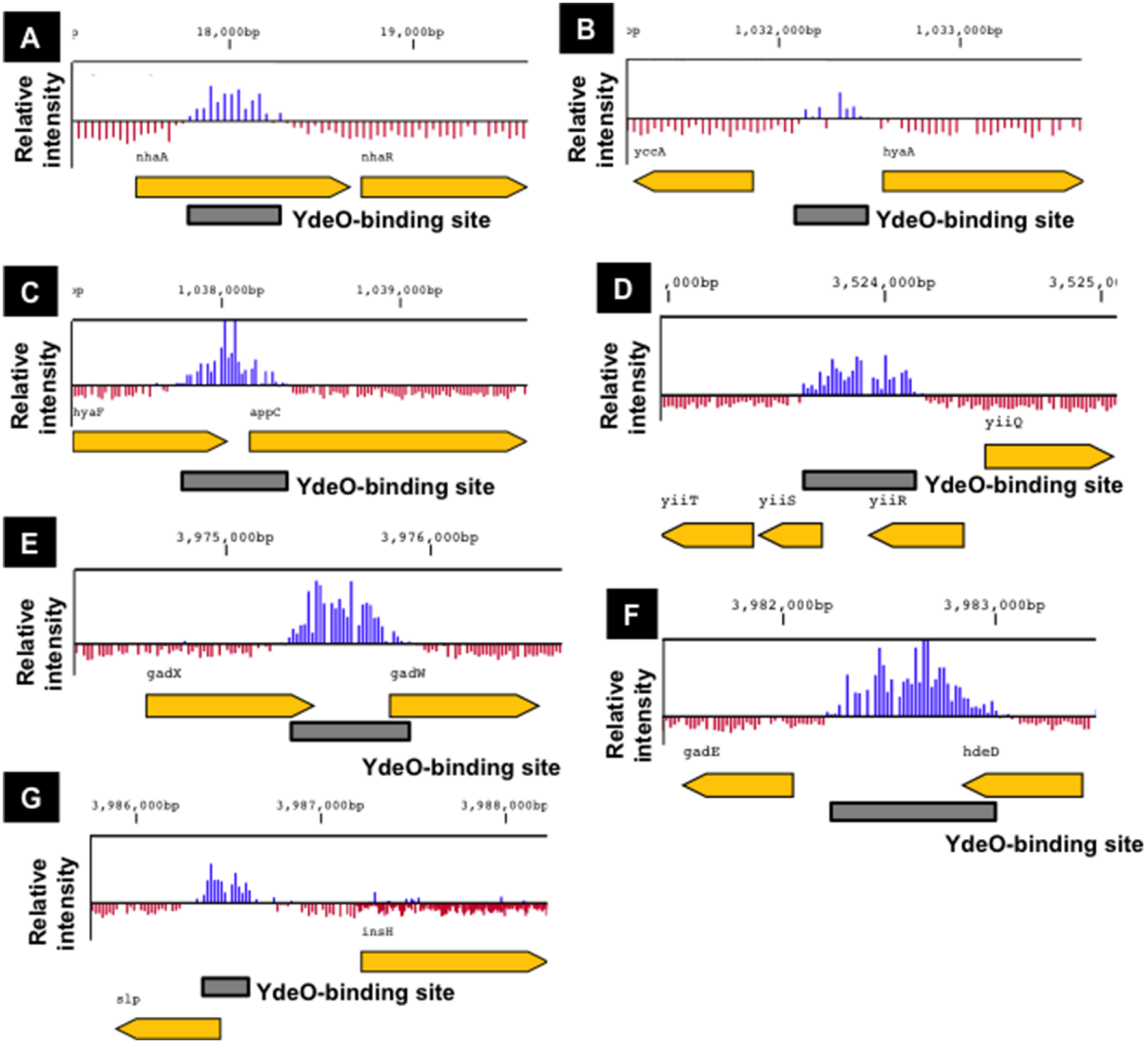
Genome-wide regulation of the *Escherichia coli* YdeO protein. Location of YdeO binding sites. The panel shows detailed YdeO binding data from ChIP-chip experiments at the *nhaR* (A), *hyaA* (B), *appC* (C), *yiiS* (D), *gadW* (E), *gadE* (F), and *slp* (G) genomic loci. The box indicates the YdeO-binding site.

To identify the genome-wide YdeO-binding sites on the entire *E. coli* genome, Sup (the whole extract DNA) and ChIP samples were each labelled and subjected to hybridization on a tiling array. Seven chromosomal regions were determined with high-level signal peaks indicating YdeO-binding, which were distinguishable from the background intensities (Fig. 1), including the *gadE*p2p3 promoters (Fig. 1F), the only known direct target of YdeO [9]. Six additional YdeO-binding sites were identified by ChIP-chip and were located within intergenic chromosomal regions. These included the intergenic spacer between *yccA* (an inner membrane protein) and *hyaA* (hydrogenase I) (Fig. 1B); the intergenic spacer upstream of *appC* (cytochrome bd-II oxidase) (Fig. 1C); the intergenic spacer upstream of the *yiiS* gene (a conserved protein) (Fig. 1D); the intergenic spacer upstream of the *gadW* gene (the *gad* operon regulator) (Fig. 1E); the intergenic spacer upstream of the *gadE* gene (the *gad* operon regulator) (Fig. 1F); and the intergenic spacer upstream of the *slp* gene (an outer membrane lipoprotein) (Fig. 1G). Although one YdeO-binding site was located inside the *nhaA* ORF another binding site was identified upstream of *nhaR* (Fig. 1A), in which the *nhaR* promoter has previously been identified [25]. Thus, all of YdeO binding sites were found in the vicinity of possible promoters (see below).

### Identification of YdeO-binding *in vitro* to the seven targets

**Figure 2:**
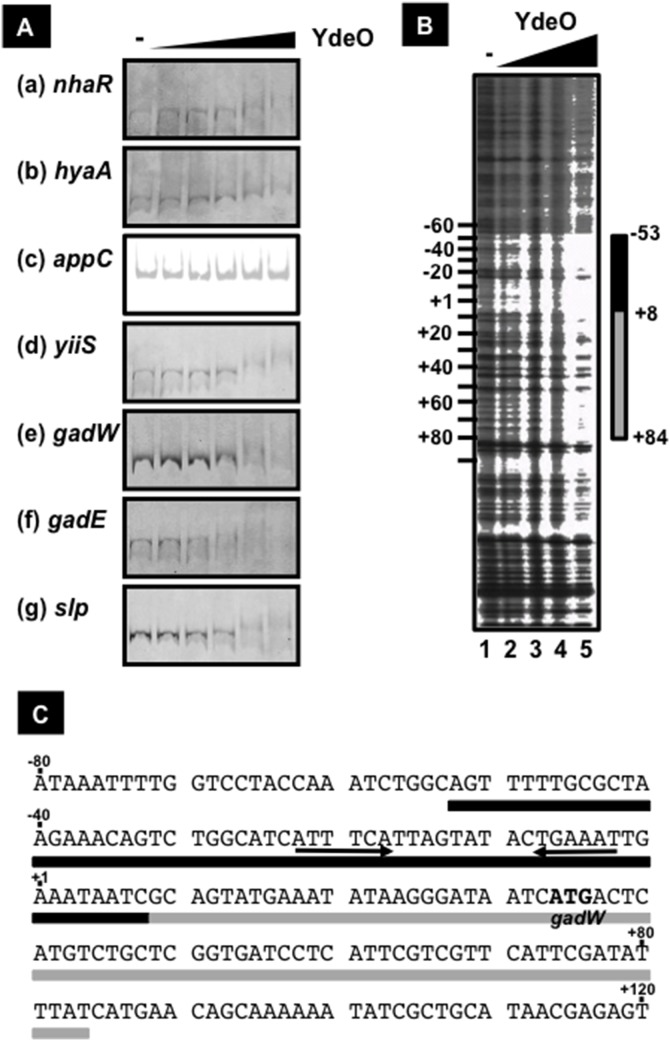
The binding of YdeO on target promoters. [A] The binding of YdeO to the target DNA, *nhaR* (a), *hyaA* (b), *appC* (c), *yiiS* (d), *gadW* (e), *gadE* (f), and *slp* (g). Probes were amplified by PCR using constructed reporter plasmids as templates and a pair of primers as the following; pLUXnhaR and a pair of NHAR-lux-F and Lux-R-FITC for *nhaR* probe; pHYAA-L and a pair of HYAA-LF and lacZ-30R-FITC for *hyaA* probe; pAPPC-L and a pair of APPC-LF and lacZ-30R-FITC for *appC* probe; pYY0503 and a pair of YIIS-LF and lacZ-30R-FITC for *yiiS* probe; pLUXgadWp and a pair of GADW-F-2 and Lux-R-FITC for *gadW* probe; pLUXgadEp and a pair of GADE-SCL-F-2 and Lux-R-FITC for *gadE* probe; and pLUXslpp and a pair of SLP-F-2 and Lux-R-FITC for *slp* probe; (Table S1). Each FITC-labeled probe (1 pmol) was incubated with YdeO protein (1, 5, 10, 25, or 35 pmol) and then DNA-YdeO complex was analyzed by native PAGE. Solid and dot lines indicate the migration of free DNA probe and DNA-YdeO complex, respectively. [B] The YdeO-binding site on *gadW* promoter. FITC-labeled probe (1 pmol) was incubated with 0 (lane 1), 0.5 (lane 2), 1.0 (lane 3), 5.0 (lane 4), or 15 (lane 5) pmol YdeO protein and then digested by DNase I. Sanger ladders are synthesized using Lux-R-FITC primer and pLUXgadWp plasmid as a template. A bar indicates the major region protected from DNase I digestion. The numbers represent the position from the transcription start site of *gadW*p1 promoter. [C] The sequence of YdeO-binding on *gadW* promoter. Black and gray bars indicate the major and minor YdeO-binding regions, respectively, as shown in [B]. The initiation codon of *gadW* coding is represented as a bold triplet. The numbers represent the position from the transcription start site of *gadW*p1 promoter.

In order to confirm the direct interaction of YdeO to the seven target sequences determined by ChIP-chip, we performed the EMSA assay. Firstly we failed to purify the YdeO protein using the pET system, because the over-expressed YdeO proteins formed inclusion bodies in *E. coli* cells. Next YdeO was over-expressed as a His-SUMO fusion, and the His-SUMO-tagged YdeO protein could be purified in soluble forms by affinity chromatography with Ni-NTA agarose (data not shown). After treatment with SUMO protease to remove the His-SUMO tag, the intact YdeO protein, however, became insoluble. Then we used this His-SUMO-tagged YdeO as the test protein. The purified His-SUMO-YdeO protein bound to the *gadE*p2p3 promoters, the only known target of YdeO (Fig. 2A-f), in good agreement with the previous report [9]. Besides the *gadE* promoter, His-SUMO-YdeO formed complexes with the *nhaR* (Fig. 2A-a), *hyaA* (Fig. 2A-b), *yiiS* (Fig. 2A-d), *gadW* (Fig. 2A-e), and *slp* (Fig. 2A-g) promoters, which were observed as a smeared band, in the presence of 10-fold molar excess of YdeO over the DNA probes. A detectable level of the YdeO-probe complex was not formed with the *appC* promoter even in the presence of 35-fold molar excess of YdeO (Fig. 2A-c). These results indicate that YdeO directly binds to at least these six sites. YdeO-DNA was detected as a smeared band in several cases, implying the cooperative binding of YdeO at the higher concentration. Since the association of YdeO with the *appC* promoter was observed only *in vivo* (see Fig. 1), this association might require another factor(s) for effective binding.

### Regulation *in vivo* of the predicted targets by YdeO: Transcriptome and RT-qPCR assays

We analyzed the alteration in the *E. coli* K-12 transcriptome caused by the over-expression of YdeO from a plasmid. *E. coli* KP7600 harboring pYY0401 (*ydeO*-*3xflag*) or the empty expression vector, pTrc99A, were grown until log phase under the same conditions used for ChIP-chip analysis, and total RNAs from these cultures were subjected to transcriptome analysis. Amongst genes downstream of a YdeO-binding site, 19 genes, (*nhaA, nhaR, hyaA, hyaB, hyaC, hyaD, hyaE, hyaF, appC, appB, appA, yiiS, yiiT/uspD, slp, dctR, gadE, mdtE, mdtF,* and *gadW*) were induced more than 3-fold by the over-expression of YdeO; while three genes, *yccA, yiiR,* and *yhiS*, were not affected in both duplicate experiments. (Table S2 and see also Table 1). These 19 genes induced by YdeO constitute a total of 7 transcriptional units, *nhaAR*, *hyaABCDEF, appCBA, yiiS-yiiT/uspD, slp-dctR*, *gadE-mdtEF*, and *gadW*. All 7 of these operons carry promoters containing YdeO-binding sites (see Fig. 2), and thus should be under the direct control of YdeO. We also examined the induction of these transcriptional units by the expression of YdeO by RT-qPCR after expression of YdeO. Transcripts of some representative genes from each operon were measured using specific pairs of the respective primers (Table S1). Transcripts were found to increase for all seven operons, *nhaAR, hyaABCDEF, appCBA, yiiS-uspD(yiiT), gadW, gadE-mdtEF,* and *slp-dctR,* in the *ydeO*-expressing cells (Table 2).

**Table 1:** Genes up-regulated by YdeO expression.

	Gene name	Transcriptoin units^a^	Wild type (Log_10_ ratio^b^)		Δ*gadE* (Log_10_ ratio^b^)	
			1st^c^	2nd^c^	1st^c^	2nd^c^
In both wild type and Δ*gadE*	*adiC*	*adiC*	1.68	1.68	1.65	1.42
	*appC*	*appCBA*	1.66	1.85	1.73	1.64
	*appB*	*appCBA*	1.71	1.69	1.71	1.60
	*appA*	*appCBA*	1.34	1.33	1.04	1.12
	*dnaK*	*dnaK-tpke11-dnaJ*	0.74	0.57	–0.01	0.46
	*dnaJ*	*dnaK-tpke12-dnaJ*	0.66	0.57	0.04	0.75
	*hyaA*	*hyaABCDEF*	1.46	1.54	1.87	1.17
	*hyaB*	*hyaABCDEF*	1.61	1.50	2.16	1.11
	*hyaC*	*hyaABCDEF*	1.46	1.53	1.84	1.20
	*hyaD*	*hyaABCDEF*	1.22	1.29	1.57	1.03
	*hyaE*	*hyaABCDEF*	1.19	1.02	1.45	0.92
	*hyaF*	*hyaABCDEF*	1.15	1.09	1.4\5	1.09
	*ibpA*	*ibpAB*	0.65	0.50	0.09	0.64
	*katE*	*katE*	0.78	0.72	0.08	0.46
	*metK*	*metK*	0.64	0.63	0.03	0.60
	*nhaA*	*nhaAR*	0.53	0.50	0.54	0.40
	*yehX*	*osmF-yehYXW*	0.67	0.63	0.22	0.46
	*slp*	*slp-dctR*	1.99	2.15	2.41	1.79
	*dctR*	*slp-dctR*	1.85	1.16	2.08	1.82
	*thrA*	*thrLABC*	0.50	0.59	0.04	0.49
	*ybaS*	*ybaST*	1.55	1.52	1.38	0.87
	*ybaT*	*ybaST*	1.31	1.22	1.29	0.73
	*ynaI*	*ynaI*	0.77	0.74	0.70	0.63
In wild type but not Δ*gadE*	*aidB*	*aidB*	1.11	1.13	0.03	–0.31
	*blc*	*blc*	0.61	0.64	0.35	–0.27
	*cbpA*	*cbpAM*	0.57	0.53	–0.15	–0.50
	*cbpM*	*cbpAM*	0.62	0.53	–0.04	–0.47
	*dps*	*dps*	0.56	0.55	0.41	–0.18
	*elaB*	*elaB*	0.60	0.53	0.10	0.08
	*gabT*	*gabDTP*	0.52	0.52	0.18	0.11
	*gadA*	*gadAX*	2.03	2.23	–0.09	0.04
	*gadX*	*gadAX*	1.03	0.87	0.27	–0.05
	*gadC*	*gadCB*	2.18	2.13	0.05	–0.10
	*gadB*	*gadCB*	2.23	2.22	0.09	0.05
	*gadW*	*gadW*	0.51	0.50	0.15	0.02
	*hdeA*	*hdeAB-yhiD*	1.89	2.06	0.81	0.75
	*hdeB*	*hdeAB-yhiD*	1.85	2.10	1.04	0.75
	*hdeD*	*hdeD*	1.72	1.84	0.60	0.27
	*mdtE*	*mdtEF*	1.89	1.94	–0.40	–0.49
	*mdtF*	*mdtEF*	1.93	1.93	0.50	0.18
	*osmF*	*osmF-yehYXW*	0.61	0.60	0.11	0.17
	*yehY*	*osmF-yehYXW*	0.59	0.65	0.25	0.29
	*pagP*	*pagP*	0.92	0.95	–0.38	0.21
	*sufA*	*sufABCDSE*	0.93	0.77	–0.18	–0.20
	*sufB*	*sufABCDSE*	0.56	0.50	–0.25	–0.31
	*wrbA*	*wrbA-yccJ*	0.76	0.70	0.40	0.13
	*yccJ*	*wrbA-yccJ*	0.73	0.64	0.55	0.14
	*ycaC*	*ycaC*	0.59	0.58	0.28	0.12
	*yfcG*	*yfcG*	0.68	0.61	0.18	0.05
	*ygaM*	*ygaM*	0.63	0.62	0.12	–0.03
	*yhiM*	*yhiM*	1.98	1.75	0.33	0.23
	*yjjU*	*yjjUV*	0.58	0.63	–0.39	–0.41
	*yjjV*	*yjjUV*	0.58	0.62	–0.11	–0.28

a Transcriptional unit is represented according to the Regulon DB (http://regulondb.ccg.unam.mx/).

b The processed intensity was calculated by Agilent Future Extraction. More than 0.5 of log ratio in WT.

c Experiment was independently performed twice (each ratio is shown as 1st and 2nd).

**Table 2:** Induction of gene expression by YdeO.

Gene name	Log_10_ ratio
*nhaA*	0.62±0.02
*nhaR*	0.31±0.05
*yccA*	–0.04±0.04
*hyaA*	0.72±0.20
*hyaF*	1.66±0.04
*appC*	2.64±0.04
*appA*	1.72±0.03
*yiiT/uspD*	0.70±0.03
*yiiS*	0.72±0.02
*gadW*	0.39±0.04
*gadE*	2.91±0.06
*mdtF*	2.36±0.07
*slp*	2.86±0.04
*dctR*	1.47±0.25

Ratio (*ydeO* ^+^/vector) ± SEM is determined by RT-qPCR as described in Materials and methods.

We also measured the level of mRNAs in the *ydeO*-deficeint mutant, but detectable differences were not found for the mRNA from YdeO-target genes between the wild-type and the *ydeO* mutant. Transcript of *yccA*, an opposite direction gene from *hyaA,* was also not affected in the presence and absence of the YdeO-expressing plasmid (Table 2). Although the *yiiS* and *uspD* genes, encoding conserved proteins with unidentified function, were expressed even without the over-expression of YdeO, their expressions were further increased after YdeO expression. These results altogether indicate that YdeO plays a role as a positive regulator for expression of all seven operons, *nhaAR*, *hyaABCDEF, appCBA, yiiS-yiiT/uspD, gadW, gadE-mdtEF*, and *slp-dctR*.

### Regulation *in vivo* of the predicted targets by YdeO: Reporter assay

**Figure 3:**
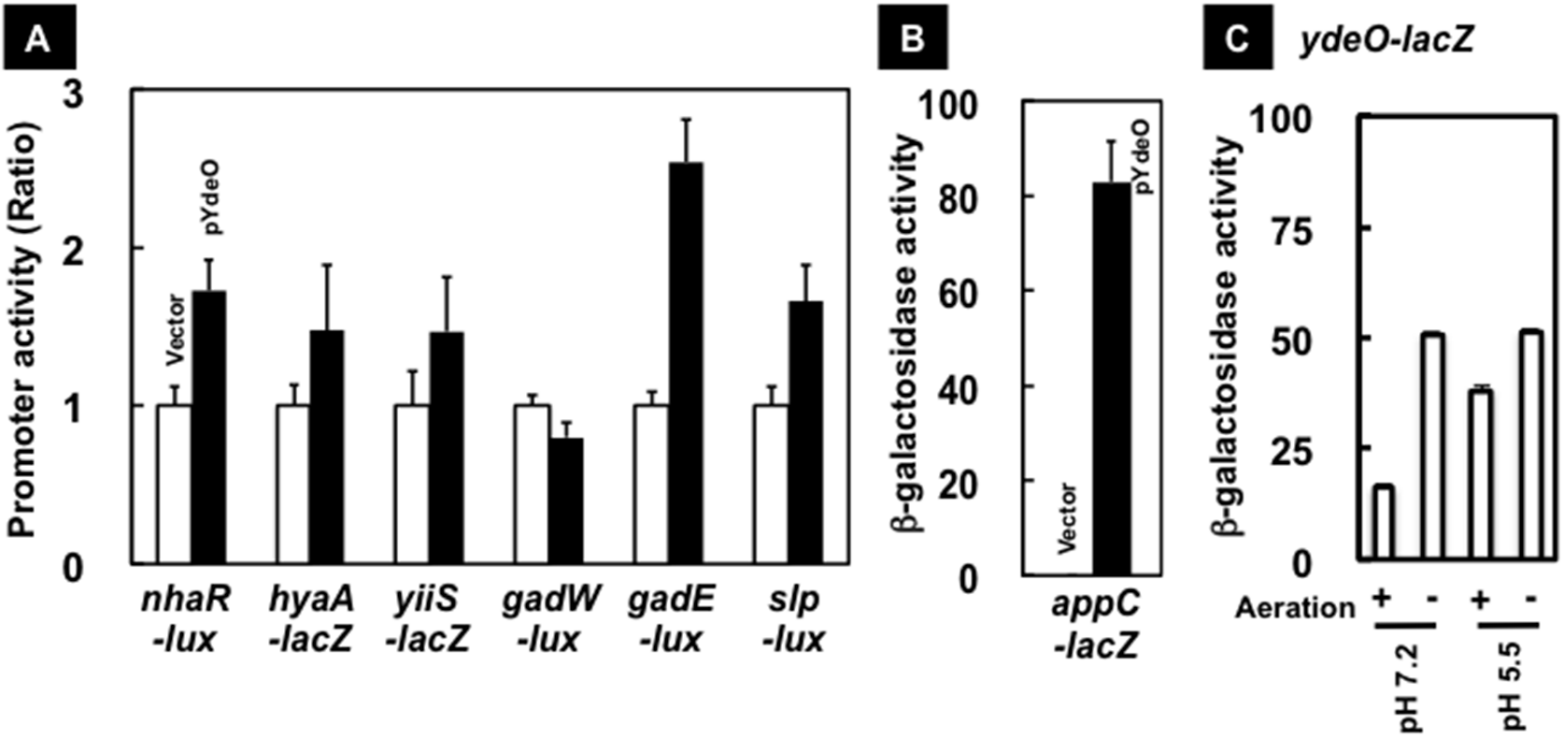
Reporter assays for transcriptional regulation by YdeO. [A] YdeO-expression induces the expression of target promoters. YY0201/pLUXnhaR (*nhaR-lux*), HYAA-JL (*hyaA-lacZ*), YY1101 (*yiiS-lacZ*), YY0201/pLUXgadWp (gadW-lux), YY0201/pLUXgadE (*gadE-lux*), and YY0201/pLUXslpp (*slp-lux*), and were transformed with pTrc99A (vector, white bar) and pYdeO (*ydeO*, black bar). Transformants grew until logarithmic phase and β-galactosidase and luciferase activities of cultures were measured as described in Materials and methods. The data show the average of independent eight experiments with standard deviation as the ratio of a vector-transformant. [B] APPC-JL (*appC-lacZ*) was transformed with pTrc99A (vector) or pYdeO (*ydeO*). Transformants grew until logarithmic phase and β-galactosidase was measured as decribed in [A]. The data show the average of independent eight experiments with standard deviation as the Miller unit. [C] The *ydeO* expression induced under anaerobic conditions. The activity of *ydeO* promoter was measured in YY0101 growing in LB medium with pH 7.2 and 5.5 under aerobic (+) and anaerobic (−) conditions at logarithmic phase.

To confirm the positive role of YdeO on expression of the newly identified target promoters, we performed the reporter assay using the *lacZ* reporter [15] and *lux* reporter [16] systems. The translation fusions, *hyaA-lacZ, appC-lacZ,* and *yiiS-lacZ,* on the pRS552 derivative plasmids were introduced at the attachment (att) site of the *E. coli* YY0201 chromosome using the λRS45 phage, resulting in isolation of HYAA-JL (*hyaA-lacZ*), APPC-JL (*appC-lacZ*), and YY1101 (*yiiS-lacZ*). Three *E. coli* lysogens containing *hyaA*, *appC*, and *yiiS* translational *lac* fusions in their chromosomes were transformed with either the YdeO-expression plasmid or the vector plasmid. The β-galactosidase activities in these transformants were measured in log-phase (Fig. 3A). YdeO-expression was found to induce the expression of all these test promoters, *hyaA-lacZ, yiiS-lacZ*, and *appC-lacZ* (Fig. 3A and B). In the cases of *hyaA-lacZ* and *yiiS-lacZ*, the promoter activity increased approximately 1.5 fold upon expression of YdeO. The detectable level of expression was not observed for *appC-lacZ* in the absence of YdeO expression but a high-level of *appC-lacZ* activity was detected upon expression of YdeO (Fig. 3B). The result indicates YdeO has a positive role in activation of the *appC*, *hyaA*, and *yiiS* promoters, in agreement with the observation by transcriptome and RT-qPCR (see above).

The *nhaR*, *slp, gadE* and *gadW* promoters were too weak for quantitation by the LacZ reporter system, so we then employed the more sensitive Lux reporter system. The *lux* reporter plasmids of four transcription fusions, *slp-lux, gadE-lux* and *gadW-lux* (kindly provide by Peter Lund [16]) and the *nhaR-lux* plasmid [constructed in this study], were introduced into YY0201 *E. coli* carrying either the vector plasmid or the YdeO-expression plasmid. The expression of *nhaR-lux*, *slp-lux,* and *gadE-lux* was found to be activated in the presence of the YdeO-expressing plasmid (Fig. 3A), indicating that YdeO is also a positive regulator for these promoters. Recently RNA-seq analysis indicated the presence of a novel *nhaR* promoter inside the coding region of *nhaA* [25]. The binding site of YdeO is located upstream of this putative promoter (see above). Accordingly the constructed *nhaR-lux* reporter plasmid containing this novel *nhaR* promoter was also activated in the presence of YdeO expression (Fig. 3A).

The expression level of *gadW-lux* stayed unaltered with and without the YdeO-expression plasmid. It is inconsistent with the RT-qPCR result that the mRNA level of *gadW* increased in the presence of YdeO expression as detected by RT-qPCR (Table 2). This apparent disagreement might be due to translational inhibition of *gadW-lux* by the anti-sense RNA of *gadW*, named *gadY*, encoded in the *gadW*-lux plasmid.

### Recognition sequence of YdeO transcription factor

**Figure 4:**
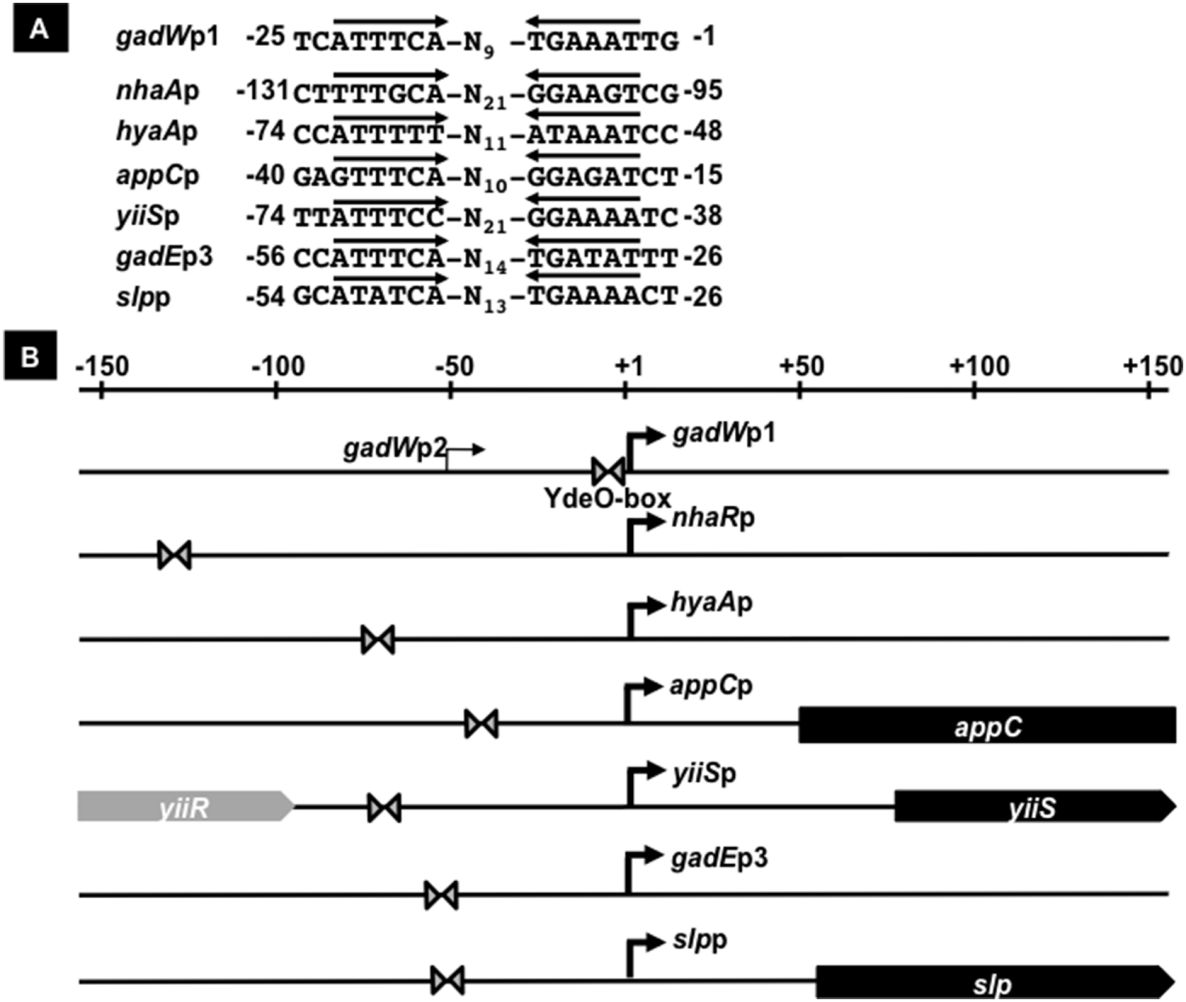
The characterization and location of YdeO-box on target promoters. We examined the conservation of the inverted repeat across seven YdeO-binding regions detected *in vivo* by ChIP-chip analysis, 131-bp on *nhaR* promoter, 216-bp on *hyaA* promoter, 139-bp on *appC* promoter, 217-bp on *yiiS* promoter, 181-bp on *gadW* promoter, 241-bp on *gadE* promoter, and 145-bp on *slp* promoter. [A] The panel shows the DNA sequence, containing the identified hexa-mer repeat (YdeO-box). The YdeO-box identified in all of promoters located on seven binding sites of YdeO. The number indicates the distance from each transcription start site (RegulonDB [http://regulondb.ccg.unam.mx]). [B] Organization of the promoters controlled by YdeO is shown. The locations of a hexamer of YdeO-binding sites (triangle) at relative positions from the transcription initiation site (solid arrow) are shown for the promoters. The filled bars represent open reading frames of the target genes.

To identify the YdeO-binding sequence, we performed DNase I footprinting of the *gadW* promoter with increasing concentrations of YdeO. At low protein levels, YdeO protected the region from –53 to +8 of the *gadW* promoter (Fig. 2B, lanes 2–4). In the presence of 15-fold molar excess of YdeO, the protected region by YdeO expanded from –53 to +84 of the *gadW* promoter possibly due to protein-protein interaction (Fig. 2B, lane 5) in agreement with the smeared band formation observed by EMSA (see above). Within the core YdeO-binding region, the inverted repeat of hexa-nucleotides, 5′-ATTTCA-3′, was identified (see Figs. 2C and 4A).

Using this YdeO-box sequence, we searched for this inverted repeat within the seven YdeO-binding regions detected by *in vivo* by ChIP-chip analysis, and identified this inverted repeat sequence of all the YdeO-binding regions at various positions between –131 to –1 with respect to the transcription start site (Fig. 4). The length of spacer between the 5′-ATTTCA-3′ hexa-nucleotide sequence ranges from 9 to 21 nucleotides (Fig. 4A). Recent studies show that YpdB and YehT bind to the direct repeat of their specific sequence separated by a 9- and 13-bp spacer, respectively, in *E. coli* [26], [27]. Previous work shows that the spacer length of the specific DNA binding region is diverse for the *E. coli* transcription factor CpxR [28]. Therefore, we have denoted the inverted repeat as the YdeO-box (Fig. 4).

### Induction of NhaR, GadE, and GadW by YdeO

Four transcription factors, the LysR-type NhaR, the LuxR-type GadE, and the AraC-type GadW, were found to be under the direct control of YdeO (see Figs. 1–4). NhaR is an activator of a sodium/proton antiporter gene [29] and both GadE and GadW are involved in regulation of the genes for glutamate-dependent acid resistance system [8], [9]. In addition to these three transcription factors, the gene encoding the CadC-like transcription factor DctR is located downstream of the *slp* gene which codes for a starvation lipoprotein, and is considered to be co-transcribed with the *slp* gene. DctR is involved in protection against metabolic endproducts under acidic conditions [30]. To examine the involvement of YdeO in control of expression of the three transcription factors, the cellular level of these proteins in *E. coli,* with or without the YdeO-expressing plasmid, were measured by Western blotting assay.

**Figure 5:**
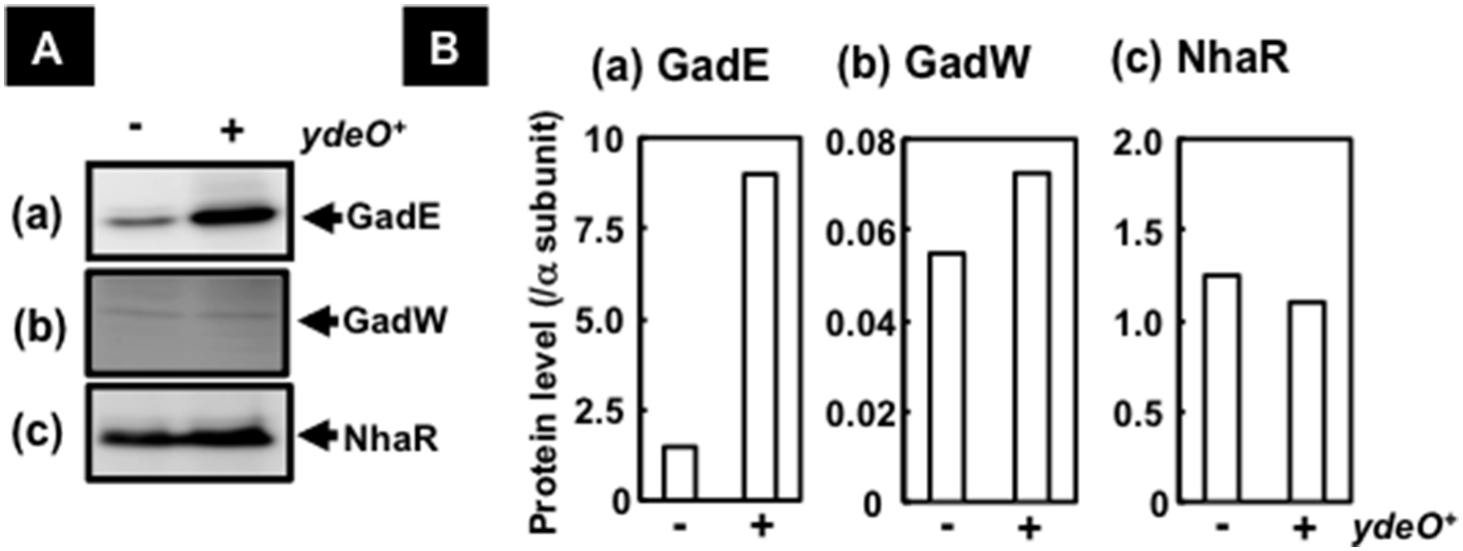
Induction of NhaR, GadE, and GadW by YdeO in *E. coli*. YY5002 (*gadE*-*3xflag*) (a), YY5003 (*gadW*-*3xflag*) (b), and BW25113, (c) harboring pTrc99A (−) or pYdeO (+), were grown in LB medium until logarithmic phase. After centrifugation the lysate solution was prepared in lysis buffer containing 8 M urea by sonication. The lysates were subjected to western blotting as described in Materials and Methods. Anti-FLAG (SIGMA) and anti-NhaR (Lab preparation) were used for detection of GadE-3xFLAG/GadW-3xFLAG, and NhaR, respectively [A]. The amounts of GadE-3xFLAG, GadW-3xFLAg, and NhaR were represented as the ratio of level of RNA polymerase-α subunit, detected by anti-α (Neoclone) [B].

To perform the Western blotting assay of GadE and GadW using the anti-FLAG antibody, we constructed *E. coli* strains YY5002 and YY5003 including *3xflag* tag at the 3′-terminal end of *gadE* and *gadW*, respectively, on the *E. coli* chromosome. The YdeO-expression plasmid, pYdeO (*ydeO*), and the empty expression vector pTrc99A were transformed into these *E. coli* strains and the transformants were grown in LB medium until log phase. The whole-cell lysates were prepared, and subjected to Western blotting assay by using anti-NhaR, anti-FLAG, and anti-RpoA for detection of NhaR, GadE-3xFLAG and GadW-3xFLAG, and RNA polymerase α subunit, respectively. All transformants with or without the YdeO-expressing plasmid retain approximately a constant amount of the α subunit of RNA polymerase (data not shown). The level of GadE increased in the YY5002 harboring the YdeO-expression plasmid, supporting the prediction that the *gadE* gene is under the direct and positive control of YdeO. However, we failed to detect NhaR and GadW even in the presence of YdeO expression (Fig. 5).

### Search for the whole set of genes regulated by YdeO > GadE

**Figure 6:**
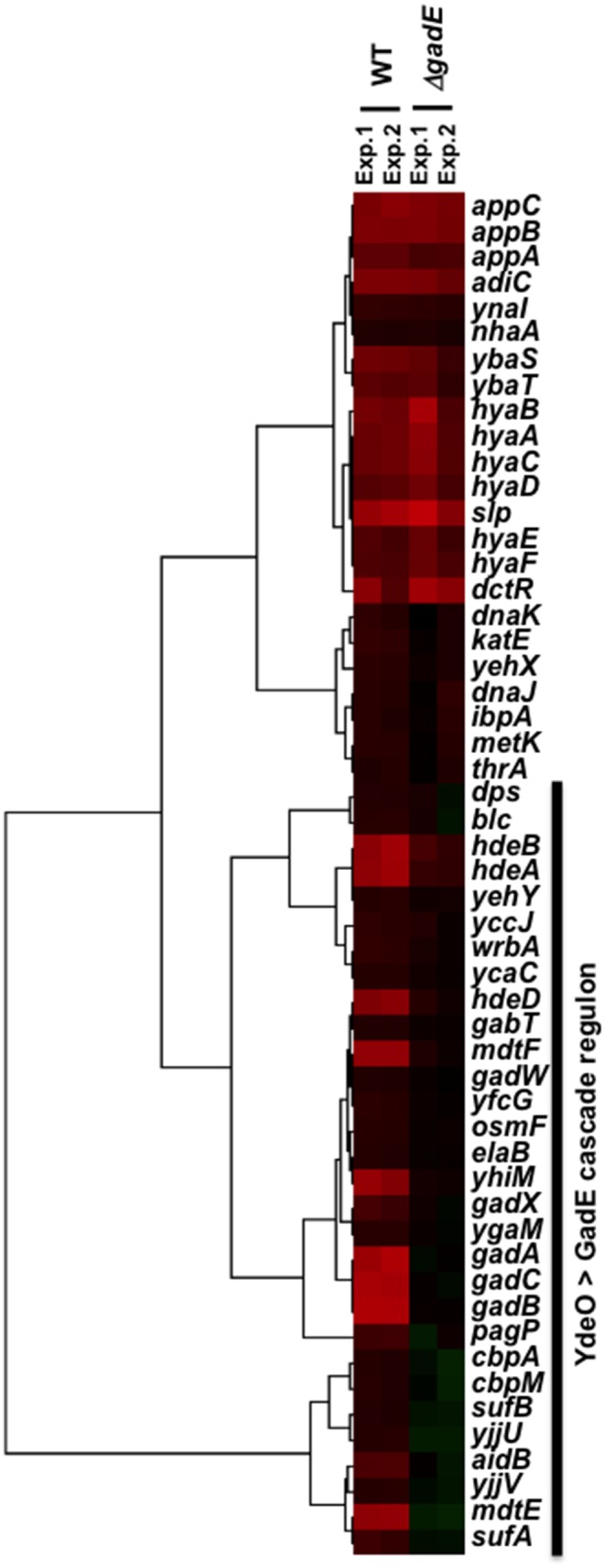
YdeO > GadE cascade regulation in *E. coli*. This shows the clustering pattern of expression of genes induced by the *ydeO*-expression in the parent (KP7600) and *gadE*-deficient mutant (JD25278) by Cluster 3.0 (http://bonsai.hgc.jp/~mdehoon/software/cluster/software.htm).

To obtain the gene expression profile of the YdeO > GadE cascade, we performed a transcriptome assay. *E. coli* wild-type KP7600 and *gadE*-deficient JD25278 harbouring pTrc99A and pYY0401 (*ydeO-3xflag*) were incubated in LB medium at 37°C with shaking until log phase and total RNA from these cultures was subjected to transcriptome analysis under standard experimental conditions as described in Materials and methods. The results revealed that a total of 106 genes were markedly affected by YdeO expression in the wild-type and included 53 up- and the same number of down-regulated genes (Tables S2 and S3). Among the 53 genes up-regulated by YdeO expression, clustering analysis showed 23 genes were induced in both the parent strain and the *gadE*-deficient mutant and 30 genes induced in the wild-type but not the *gadE*-deficient mutant (Fig. 6). The observed alteration of the transcriptome profile caused by deletion of the *gadE* gene was similar to that reported by Masuda and Church [31]. Genes induced in both strains are organized into a total of 12 transcriptional units (Table 1), including five transcription units, *hyaABCDEF, appCBA, slp-dctR*, and *nhaAR*, that are under the direct control of YdeO (see above). On the other hand, the rest of the 30 up-regulated genes forming 21 transcription units were induced in the wild-type but not in the *gadE*-deficient mutant (Table 1), indicating that these 21 transcription units are under the direct control of GadE but the indirect control of YdeO. This set of 21 transcription units includes the hitherto identified GadE targets, *gadA*, *gadB*, and *gadC* [9]. On the other hand, detectable change was not observed in the transcription pattern between the parent strain and the *gadW*-deficient mutant, consistent with the lack of YdeO-dependent GadW expression under the conditions herein employed (Figs. 3 and 5). The *yehX* gene was induced by the YdeO-expression plasmid in both the parent strain and the *gadE* mutant but the *osmF* and *yehY* genes, and parts of *osmF-yehYXW* transcription unit, were not induced in the *gadE* mutant (Fig. 6 and Table 1), implying that GadE activates the known promoters located at the upstream of *yehX* which is possibly activated by YdeO.

### Physiological roles of YdeO in response to environmental stresses

The level of translational control of the YdeO regulator itself was analyzed using a reporter assay with the *ydeO-lacZ* fusion. In *E. coli* YY0101 (*ydeO-lacZ*) grown under aerobic conditions, β-galactosidase activity from *ydeO-lacZ* increased two-fold under the acidic condition of pH 5.5 compared with pH 7.0 (Fig. 3C). Interestingly the high level of *ydeO-lacZ* was detected in both pH 5.5 and 7.0 when *E. coli* were grown under anaerobic conditions (Fig. 3C), implying that YdeO plays a role in *E. coli* respiration under anaerobic conditions, such as in the animal intestine. Previously, we identified that the transcription of *ydeO* is induced by exposure to ultraviolet light via the two-component system EvgSA two-component system [11]. In agreement with this finding, *ydeO* expression was not induced in the *evgA*-defective mutant under both acidic and anaerobic conditions (data not shown).

## Discussion

### The YdeO regulon

**Figure 7:**
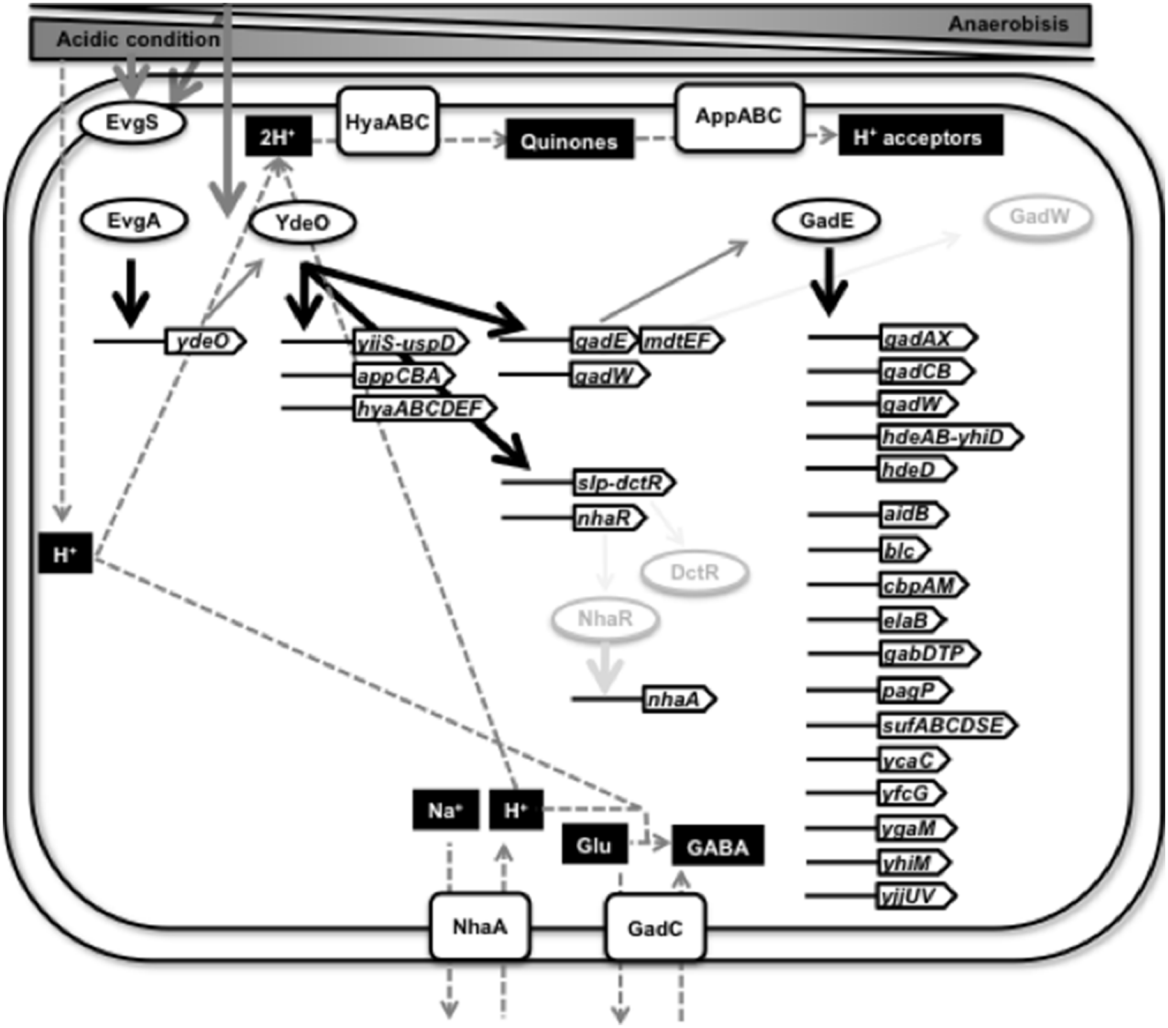
EvgAS > YdeO > DctR/NhaR/GadE/GadW regulatory network in *E. coli*.

Here we have identified a total of seven YdeO-binding sites on the *E. coli* genome using ChIP-chip and transcription analyses *in vivo*. The EMSA experiments showed that purified YdeO also binds *in vitro* to these six sites (see Fig. 2). The reporter and RT-qPCR assays indicated that all of the promoters located downstream of these YdeO-binding sites are activated by YdeO (see Table 1 and Fig. 3). The hexa-nucleotide repeat 5′-ATTTCA-3′, which we have named the YdeO box, is conserved in all of YdeO-binding sites we identified experimentally (see Fig. 4). Even though this YdeO-box like sequence exists within the *appC* promoter, which is located immediately downstream of a YdeO-binding site (see Fig. 1), the binding *in vitro* of YdeO to the *appC* promoter probe was not high (see Fig. 2), implying that an as yet unidentified additional transcription factor or DNA secondary structure is needed for efficient binding of YdeO to the target promoter. Since the *appC* promoter is transcribed *in vivo* by RNA polymerase containing the RpoS sigma factor and is induced by AppY [32], [33], one possibility is that AppY and/or RpoS sigma are required for the efficient binding of YdeO to the *appC* promoter. Thus, we conclude that YdeO is a positive regulator for transcription of operons controlled by seven promoters, the *nhaR* promoter, *hyaA* promoter, *appC* promoter, *yiiS* promoter, *slp* promoter, *gadE* promoter, and *gadW* promoter (Fig. 7).

### Transcription cascade: EvgSA > YdeO > NhaR, GadE, GadW

*E. coli* responds to temporary low pH using the glutamate-dependent acid resistant system, which involves two complex regulatory systems: EvgAS > YdeO > GadE; and Crp> RpoS > GadX > GadW [9], [12]. In this study, we showed that YdeO directly regulates the expression of three transcription factor genes, *nhaR*, *gadE,* and *gadW* (see Fig. 7), proving the novel transcription cascade: EvgAS > YdeO > NhaR/GadE/GadW.

YdeO not only plays a regulatory role in positive feedback loop of EvgAS > YdeO > GadE pathway, but also a positive role in the GadXW pathway, thereby linking the GadE- and GadXW-pathways for acid resistance. The GadXW circuit is believed to function during stationary phase. YdeO-overexpression induced GadE-dependent transcription of the *gadW* gene but GadW protein was not detected in growing *E. coli* cell (Fig. 6), suggesting that stationary phase specific factors are required for GadW.

Transcriptome analysis identified the set of genes directly regulated by YdeO or indirectly through the YdeO > GadE cascade (Table 2; see Fig. 7 for the summary model). GadE induced by YdeO stimulated the transcription of *hdeAB-yhiD, hdeD, gadAX, gadCB, mdtEF, gadW*, and *yhiM* as well as those previously reported promoters [9], [34], [35]. The GAD cluster including *hdeAB-yhiD, hdeD, gadAX, gadCB, gadE, mdtEF,* and *gadW,* is necessary for glutamine-dependent acid resistance [9], [34]. Recently the *yhiM* gene was reported to be essential for growth at pH 2.5 and is necessary for glutamine- and lysine-dependent acid resistance, but is not required for arginine-dependent acid resistance [36]. In addition of these operons, the YdeO > GadE cascade induced a total of 19 operons including *aidB, blc, cbpAM, elaB, gabDTP, pagP, sufABCDSE, ycaC, yfcG, ygaM,* and *yjjUV* (see Table 1), of which the *yfcG* gene encodes a disulfide reductase [37] and the *sufABCDSE* operon encodes the complex biosynthetic machinery for iron-sulfur clusters in several enzymes which have critical cysteine residues [38], suggesting a relationship between the function of YdeO and cysteine metabolism.

### The physiological role of the YdeO regulon

In addition of the hitherto-identified target *gadE*, we have identified a total of seven operons belonging to the YdeO regulon. The expression of *ydeO* is induced under acidic conditions (see Fig. 3C). In good agreement, the *gadE* operon encodes the master activator for expression of *gadA* and *gadBC*, which are involved in the glutamate-dependent acid resistance system which works for consumption of intracellular protons by glutamate decarboxylation. In addition to acid conditions, the expression of *ydeO* is also induced under anaerobic growth in both neutral and acid conditions. Two YdeO-regulated targets, *hyaABCDEF* and *appCBA,* encode a hydrogenase and a quinone oxidase, respectively, both being involved in bacterial respiration. The HyaABC complex oxidizes dihydrogen to two protons, following release of them to the outside of the membrane, and donation of the electrons to the quinone pool. The AppBC complex donates electrons taken by a quinone to intracellular oxygen, consuming an intracellular proton per electron (Fig. 7), resulting in H_2_O production via oxygen [39]. Thus, the *hyaABCDEF* operon contributes to the consumption of the intracellular proton while the *appCBA* operon contributes to the utilization of reduced quinone. Taken together, these physiological systems activated by YdeO stimulate stress response and respiration. These findings also suggest that YdeO activated genes play an important role in primary adaptation, which enables the cell to colonize animal intestines by contributing to adaptation to acidic conditions in the stomach and to anaerobic conditions in the intestine.

## Supporting Information

Table S1 **Bacterial strains, phage, plasmids, and oligonucleotides used in this study.** *E. coli* K-12 derivatives used in this study were indicated with characterizations. The used bacteriophage and plasmids were also shown. Oligonucleotides were represented with DNA sequences.
(DOCX)

Table S2 **The genes affected by expression of** ***ydeO*** **gene in** ***E. coli*** **KP7600.** Transcriptome analysis was performed using total RNAs from KP7600 harboring pTrc99A (vector) and pYY0401 (*ydeO*-*3xflag*) as described in Materials and methods. The *E. coli* Gene Expression Microarray microarray 8×15 K (Agilent) hybridized by the fluorescent cDNAs was scanned with an Agilent G2565CA microarray scanner Ver. 8.1, the intensities of both Cy3 and Cy5 were quantified by Feature Extraction Ver. 8.1, and then, the Cy5/Cy3 ratios were calculated from the normalized values.
(XLSX)

Table S3 **The genes affected by expression of** ***ydeO*** **gene in the** ***gadE*** **-deficient** ***E. coli*** **mutant.** Transcriptome analysis was performed using total RNAs from JD25278 (*gadE*::mini-Tn10) harboring pTrc99A (vector) and pYY0401 (*ydeO*-*3xflag*) as described in Table S2. The intensities quantified by Feature Extraction Ver. 8.1 and the Cy5/Cy3 ratios were represented.
(XLSX)
