# Supplementary material for: Characterization of the YdeO Regulon in Escherichia coli
Source: PLoS One. 2014 Nov 6;9(11):e111962. doi: 10.1371/journal.pone.0111962 (PMC4222967; doi:10.1371/journal.pone.0111962)
Supplement: Table S1 — Bacterial strains, phage, plasmids, and oligonucleotides used in this study. E. coli K-12 derivatives used in this study were indicated with characterizations. The used bacteriophage and plasmids were also shown. Oligonucleotides were represented with DNA sequences. (DOCX) [file pone.0111962.s001.docx]

Table S1. Bacterial strains, phage, plasmids, and oligonucleotides used in this study.

Name Characterization Reference

***E. coli* strains**

BW25113 F^-^ *lacl^q^ rrnB3 lacZ4787 hsdR514(araBAD)567 DE(rhaBAD)568 rph-1* Haldimann & Wanner, 2001

KP7600 F^-^ *lacI^q^ lacZΔM15 galK2 galT22 λ^-^ in (rrnD-rrnE)1* Miki et al., 2008

W3110 typeA Wild type, complete σ set Jishage & Ishihama, 1997

JD25278 KP7600, *gadE*::mini-Tn10 (Km^r^) Miki et al., 2008

YY0201 BW25113, *ΔydeO* Yamanaka et al., 2012

APPC-JL YY0201, λ*appC-lacZ* (Km^r^) This study

HYAA-JL YY0201, λ*hyaA-lacZ* (Km^r^) This study

YY0101 BW25113, λ*ydeO-lacZ* (Km^r^) Yamanaka et al., 2012

YY1101 YY0201, λ*yiiS-lacZ* (Km^r^) This study

YY5001 BW25113, *ydeO*-*3xflag* (Km^r^) This study

YY5002 BW25113, *gadE*-*3xflag* (Km^r^) This study

YY5003 BW25113, *gadW*-*3xflag* (Km^r^) This study

**Phage**

λRS45 *bla'-lacZ' imm21 ind* Simon,1987

**Plasmids**

pTrc99A *trc*-inducible promoter plasmid, Ap^r^ Amann et al., 1988

pE-SUMO SUMO fusion plasmid, Ap^r^ Life Sensors

pRS552 promoter-less *lacZ* for protein fusion plasmid, Ap^r^, Km^r^ Simon et al.,1987

pKD46 FRT-recombination plasmid, Ap^r^ Datsenko & Wanner, 2000

pSUB11 FLAG sequence and FRT sites flanking a kanamycin casette Uzzau et al., 2001

pLUX promoter-less *luxCDABE* Burton et al., 2010

pLUXgadEp pLUX, *gadE’-lux* Burton et al., 2010

pLUXgadWp pLUX, *gadW’-lux* Burton et al., 2010

pLUXslpp pLUX, *slp’-lux* Burton et al., 2010

pLUXnhaR pLUX, *nhaR-lux* This study

pAPPC-L pRS552, *appC’-’lacZ* This study

pYY0503 pRS552, *yiiS’-’lacZ* This study

pHYAA-L pRS552, *hyaA’-’lacZ* This study

pYY0401 pTrc99A, *ydeO*-*3xflag* This study

pYdeO pTrc99A, *ydeO* This study

pYdeO-SUMO pE-SUMO, SUMO-*ydeO* This study

**Oligonucleotides**

APPA-F-1 5'-GCCCGCAGTCTGGTCAGGTCGCGATTATTG-3' This study

APPA-R-1 5'-AGCAAGGTGTTCCACTGGTGTGAATCGGTG-3' This study

APPC-LF 5'-GGCTTGAATTCGTATGTCAGTGGCTGGCGG-3' This study

APPC-F-2 5'-GTGACCTGGGCTACGGCATGTTGCTCTCCC-3' This study

APPC-LR 5'-GAGCAGGATCCCAGCGCGATAAATCAATGA-3' This study

APPC-R-2 5'- CCCCTGTTGCTGCGTCGGTTGTTCACTCTGC-3' This study

DCTR-F-1 5'-ATTACCAGGGATACGATGTTCTTCACCGCG-3' This study

DCTR-R-1 5'-CCAGCTCATTGATCCGCTTAAGATTGAGGA-3' This study

GADE-F 5'-TATGAGCGACATCGTCACCCTGGGTATCACAT

CTTATTTTGACTACAAAGACCATGACGG-3' This study

GADE-F-1 5'-TCTTTTACAGGGCTTTTGGCAGTTGAAAGA-3' This study

GADE-R 5'-GCCGTTCTGCCAACAGTTCCTGCCAGCATTCG

GGCACATATGAATATCCTCCTTAG-3' This study

GADE-R-1 5'-CTAAAAATAAGATGTGATACCCAGGGTGAC-3' This study

GADE-SCL-F-2 5'-TTGGGGATCGGAATTCCCCGGTTGTCACCCGGATCATAGT-3' This study

GADW-F 5'-GCCACATCAGTTTGCGCAACATTCGCCAGGTA

CCTTTTCCGACTACAAAGACCATGACGG-3' This study

GADW-F-1 5'-TAATTAACCAGTCACCCCCCATGCCGCTGG-3' This study

GADW-F-2 5'-CCCGGCATAGGGGACCGGGAAGAGGATAGT-3' This study

GADW-R 5'-TCGTATGAATTGTCAGCTTAATAATCCCAAAC

ATGCCGAGCATATGAATATCCTCCTTAG-3' This study

GADW-R-1 5'-GGTACCTGGCGAATGTTGCGCAAACTGATG-3' This study

HYAA-F-1 5'-GGCTGCCGCCGGAACCCAGGCGGAAGAAGT-3' This study

HYAA-LF 5'-CCCCAGGATCCGGCCAAAAGAAAAATAGGT-3' This study

HYAA-LR 5'-GCCGGGGATCCCCCTGACGCCGCATGGCCT-3' This study

HYAA-R-1 5'-GGAGGAACAGGCGTTATAGGTGGTAGGCCC-3' This study

HYAF-F-1 5'-TTCCATCTGCTGGGGCCAGGAACGCAACCG-3' This study

HYAF-R-1 5'-TGCGCCAACGGTAGGCCATTCATCAGGCCA-3' This study

LacZ 30R 5'-GCTGCAAGGCGATTAAGTT-3' Lab stock

lacZ-30R-FITC FITC-5'-AGGGTTTTCCCAGTCACGACGTTGTAAAAC-3' This study

Lux-R 5'-GGCAGGTAAACACTATTATCACC-3' This study

Lux-R-FITC FITC-5'-GGCAGGTAAACACTATTATCACC-3' This study

MDTF-F-1 5'-GGGAGATCTACCGCCAGTTCTCCATCACGC-3' This study

MDTF-R-1 5'-CCGACACGTTCAGACCACGGCTTGAGACTG-3' This study

NHA-lux-R 5'-ACTAACTAGAGGATCACGCCCGTGCGGCGTGCACCACACA-3' This study

NHAR-lux-F 5'-TCGTCTTCACCTCGATTATGGATAAATGACGCGCTGATGG-3' This study

NHAA-SF-2 5'-GTTGGCGTGGTGTTGTGGACTGCGGTGTTG-3' This study

NHAA-SR-2 5'-CCAATTACCGCCGAAGAGATAGAACCGACC-3' This study

NHAR-F-1 5'-CCAGCGAGCTGGGAGAACTGGTCTATCGCT-3' This study

NHAR-R-1 5'-CGCCGAGGATTTCTACGTTTAATCCCTGGG-3' This study

SLP-F-1 5'-GCTTGCCGCATGTAGTTCAATTCCGCAAAA-3' This study

SLP-F-2 5'-CGCTTATCAAATCATTACCAGAATATAATA-3' This study

SLP-R-1 5'-GACCAGCTCAGGTGTTACCTGACTCACCGC-3' This study

SUMO forward 5'-TGGTGAGGAAATTCTTCCG-3' This study

T7 terminator 5'-CTAGTTATTGCTCAGCGG-3' This study

Trc99A-F 5'-TTGCGCCGACATCATAACGGTTCTGGCAAA-3' This study

Trc99A-R 5'-ACCACCGCGCTACTGCCGCC-3' This study

YCCA-F-1 5'-TACGGTGCTGATGCTGCCATCTCCGGGTCT-3' This study

YCCA-R-1 5'-GCTCAGCAGGCTGACGAAGATGTTGTACAG-3' This study

YDEOF-1 5’-GGGTATGAATACGGGCAACACGATGAATGCT

TTAGCTATTGACTACAAAGACCATGACGG-3’ This study

YDEOF-2 5'-GAGATGAAATTCTCGTTTGTTCTGTTA-3' This study

YDEOR-1 5’-ATGCAGAATTGAGATCATAAATAATCATGCA

ACAGGCATATGAATATCCTCCTTAG-3’ This study

YDEOR-2 5'-ACTCGGGATCCAATAATCAAATAGCTAAAG-3' This study

YDEOR-3 5'-TCCAGGATCCTTACTATTTATCGTCGTCA-3' This study

YDEO-SUMO-F 5'-GAACAGATTGGAGGTATGTCGCTCGTTTGTTCTGTTATAT-3' This study

YDEO-SUMO-R 5'-ATTCGGATCCTCTAGTCAAATAGCTAAAGCATTCATCGTG-3' This study

YIIR-F-1 5'-CCATTGGCGTGGCAATTTCCGGGAATGAA-3' This study

YIIR-R-1 5'-TCGATAAACGGCACGATGAGCAAATCCGCA-3' This study

YIIS-LF 5'-TCCAGGATCCTTACTATTTATCGTCGTCA-3' This study

YIIS-F-2 5'-CGACAGAGAGGCGAATATACAGAGGTGCCC-3' This study

YIIS-LR 5'-TCCAGGATCCTTACTATTTATCGTCGTCA-3' This study

YIIS-R-2 5'-CTCGGCCTGGCATGAGAAAGTGAAGGCCGC-3' This study

16s-982 5'-CGATGCAACGCGAAGAACCT-3' Kailasan Vanaja et al., 2009

16s-1143 5'-GCCGGACCGCTGGCAACAAA-3' Kailasan Vanaja et al., 2009

References

Haldimann, A. and Wanner, B. L. (2001) Conditional-replication, integration, excision, and retrieval plasmid-host systems for gene structure-function studies of bacteria. *J Bacteriol* **183:** 6384-6393.

Miki, T., Yamamoto, Y., and Matsuda, H. (2008) A novel, simple, high-throughput method for isolation of genome-wide transposon insertion mutants of *Escherichia coli* K-12. *Methods Mol. Biol.* **416**: 195-204.

Jishage, M. and Ishihama, A. (1997) Variation in RNA polymerase sigma subunit composition within different stocks of *Escheichia coli* strain W3110. *J Bacteriol* **179**: 959-963.

Yamanaka, Y., Ishihama, A., and Yamamoto, K., (2012) Induction of YdeO, a regulator for acid resistance genes, by ultraviolet irradiation in *Escherichia coli*. *Biosci. Biotechnol. Biochem.* **76** : 1236-1238.

Simon, R. W., Hausman, F., and Kleckner, N. (1987) Improved single and multicopy lac-based cloning vectors for protein and operon fusions. *Gene* **53**: 85-96.

Amann, E., Ochs, B., and Abel, K.-J. (1988) Tightly regulated *tac* promoter vectors useful for the expression of unfused and fused proteins in *Escherichia coli*. *Gene* **69**: 301-315.

Datsenko, K. A. and Wanner, B. L. (2000) One-step inactivation of chromosomal genes in *Escherichia coli* K-12 using PCR products. *Proc. Natl. Acad. Sci. U S A.* **97:**6640-5.

Uzzau, S., Figueroa-Bossi, N., Rubino, S., and Bossi, L. (2001) Epitope tagging of chromosomal genes in *Salmonella*. *Proc Natl Acad Sci USA* **94**: 13997–14001.

Burton, N. A., Johnson, M. D., Antczak, P., Robinson, A., and Lund, P. A., (2010) Novel Aspects of the Acid Response Network of E. coli K-12 Are Revealed by a Study of Transcriptional Dynamics. *J Mol Biol* **401**: 726–742

Kailasan Vanaja, S, Bergholz, T. M., and Whittam, T. S. (2009) Characterization of the *Escherichia coli* O157:H7 Sakai GadE Regulon. *J Bacteriol* **191**: 1868-1877.
